# Optical coherence tomography image dataset of textile fabrics

**DOI:** 10.1016/j.dib.2022.108719

**Published:** 2022-11-02

**Authors:** Metin Sabuncu, Hakan Ozdemir

**Affiliations:** a Department of Electrical and Electronics Engineering, Dokuz Eylül University, Izmir, 35160, Turkey; b Department of Textile Engineering, Dokuz Eylül University, Izmir, 35160, Turkey

**Keywords:** Optical coherence imaging, Wool, Cotton, Polyester, Material classification, Deep learning, Recycling

## Abstract

We record Optical coherence tomography (OCT) images of various textile fabrics. Each textile fabric consisted of one material only: wool, cotton or polyester. We took OCT images from three different fabrics for each material type giving a total of 9 different fabrics. We scan each material at least a hundred times at different places on each surface. In order to have approximately consistent data between samples, the scans for each image were fixed to 2 mm scan length and saved in a portable network format. We divide the material data into three categories. Groups 1, 2, and 3 consisted only of cotton, wool, and polyester fabrics, respectively. These were placed in folders, becoming the labelled dataset for deep learning training classes. We publish this OCT fabric image dataset publicly. Researchers can utilize the data to train deep learning networks, test existing machine learning algorithms, or develop new systems for automated material classification and recycling.

**Specifications Table**

**Table:** 

Subject	Textile Engineering
Specific subject area	OCT imaging of textile fabrics to facilitate automated recycling via deep learning algorithms
Type of data	Image
How data were acquired	Optical coherence tomography imaging. Instruments: hardware, software Make and model and of the instruments used: Hardware: Thorlabs CAL110C1 Software: ThorImage OCT
Data format	Raw
Parameters for data collection	2-D mode of the ThorImage OCT software was switched on to take OCT- B images of the fabrics with the Thorlabs CAL110C1 device with a 2mm fixed scan length
Description of data collection	There were nine different fabrics in total. Three of the fabrics were all wool. Three fabrics consisted only of cotton fibers, and the other three fabrics consisted only of polyester. We placed each fabric in the OCT system's sample arm. We took at least a hundred scans on the fabric surface at random locations. We performed the data acquisition using ThorImage software, which recorded the image data on the computer. We used the same parameters for the data collection of all nine different substances..
Data source location	Institution: Dokuz Eylul University City/Town/Region: Tinaztepe/Buca/Izmir Country: Turkey Latitude and longitude for collected samples/data: 38°22′13.1"N 27°12′30.4″E
Data accessibility	Name of Repository: Mendeley Data Title:Optical coherence tomography image dataset of textile fabrics Link:https://data.mendeley.com/datasets/kddwp4k7ff/1 DOI: 10.17632/kddwp4k7ff.1

## Value of the Data

• The dataset contains OCT images of fabrics, which can play a crucial role in material type classification for automated recycling.
• Researchers and engineers working in textiles, material, and data science can benefit from this image dataset.
• Such OCT data may also be used in forensic sciences.
• This data can be used to train deep learning networks, test existing machine learning algorithms or help develop new deep learning systems for automated material classification.

## 1 Data Description

**Fig. 1:**
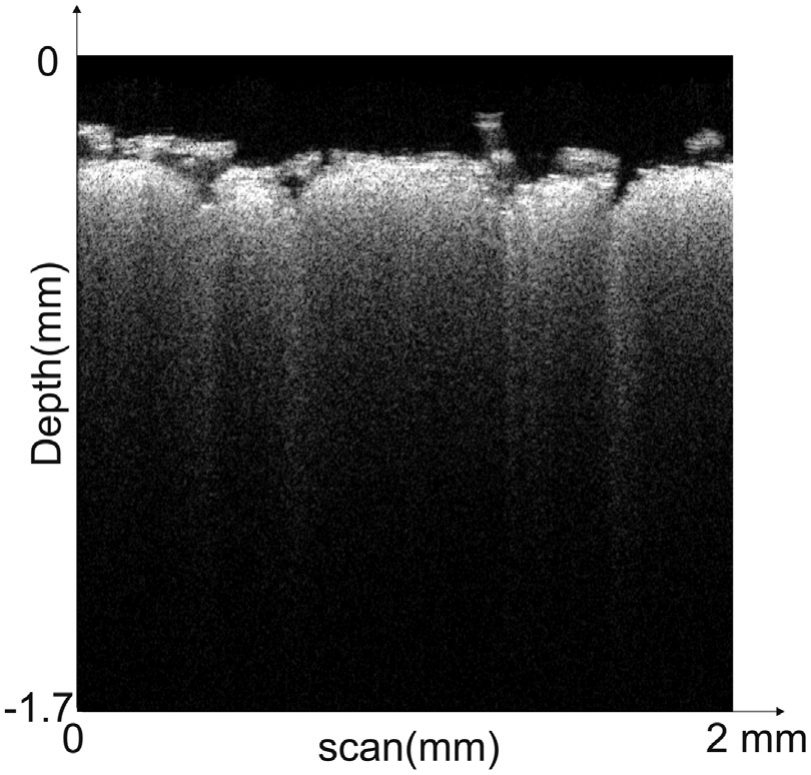
A typical OCT scan of the fabric. 2-D mode of the ThorImage OCT software was used to capture OCT- B images of the fabrics with the Thorlabs CAL110C1 device with a constant scan path of 2mm. The scan depth and length for all the images are fixed to 1.7 mm and 2 mm respectively. The image dataset consists of raw scans.

Data comprises unedited OCT scans in nine separate folders in portable network graphics format. We record OCT images taken at random locations on just one of the fabrics in its designated folder (Fig. 1). Therefore, each Wool1, Wool2, and Wool3 folder consists of OCT scans of three different fabrics made of pure wool fibers. Similarly, the Cotton1, Cotton2, and Cotton3 folders consist of OCT scans of three different fabrics made from pure cotton fibers. The folders Polyester1, Polyester2, and Polyester3 consist of OCT scans corresponding to three fabrics, that are of pure polyester origin. Each OCT image's time and date information is registered in the corresponding portable network graphic file name. The OCTImage 051502_07172020.png in the polyester1 folder corresponds to an OCT scan of a polyester fabric taken at 05:15:02 on July 17, 2020.

## 2 Experimental Design, Materials and Methods

The experiment aimed to capture OCT images of textile fabrics and generate a dataset of OCT scans corresponding to fabric materials composed of only wool, cotton, or polyester fibers [1,2]. Therefore, before we recorded the OCT scans, the fiber content of the fabrics was determined by conventional fire testing with subsequent microscopic observation [3]. We then retained only pure fabrics composed of wool, cotton, or polyester fibers for the subsequent OCT measurement. We combined three different fabrics for each fiber type and obtained nine separate samples. Each sample was placed on the sample arm and measured individually using the OCT modality.

We acquired OCT images through the Thorlabs CAL110C1 imaging system using a laser diode with a central wavelength at 930 nm [4]. The diode produces broadband photons that enable speckle-free imaging [5]. We record the OCT images via the ThorImage OCT software [6]. We placed the samples to be scanned on the sample arm. 2D image scans, known as OCT-B scans, are obtained by scanning the light beam across the sample's surface and adding the corresponding OCT-A images one at a time [7]. We set the scan path to 2 mm for all OCT-B images obtained from nine samples [8].

Each sample was placed on the sample arm, individually scanned using the OCT modality, and saved in a portable network graph format in a separate folder. This provided approximately consistent data across samples. OCT images are raw OCT-B files saved via the ThorImage software program. We upload unedited OCT images and do not do any additional image processing or filtering. Because most deep learning algorithms require at least a hundred images per class for training, we have provided 120 to 200 OCT scans per item [9], [10], [11], [12], [13], [14], [15].

## Ethics Statement

The work did not involve any human subjects or animal experiments.

## CRediT Author Statement

**Metin Sabuncu:** Conceptualization, Experiment, Data Acquisition, Writing; **Hakan Ozdemir:** Fabric preparation, Analysis, Review and Editing.

## Declaration of Competing Interest

The authors declare no conflict of interest for this article.

## Data Availability

Optical coherence tomography image dataset of textile fabrics (Original data) (Mendeley Data).
